# The work of Chinese chronic conditions: adaptation and validation of the Distribution of Co-Care Activities Scale

**DOI:** 10.3389/fpubh.2023.1091573

**Published:** 2023-04-17

**Authors:** Mingyue Zhou, Mingxin Liu, Qi Lu, Bailing Hou, Yue Yuan, Sien Pan, Huijun Zhang

**Affiliations:** ^1^School of Nursing, Jinzhou Medical University, Jinzhou, Liaoning, China; ^2^Liaoning Cancer Institute and Hospital, Shenyang, Liaoning, China; ^3^Department of Endoscopy, General Hospital of Northern Theatre Command, Shenyang, Liaoning, China; ^4^Department of Oncology, People's Hospital of Liaoning Province, Shenyang, Liaoning, China

**Keywords:** primary health care, chronic disease management, co-care activity, patient experience, adaptation, validation, patient preference, patient-centered

## Abstract

**Purpose:**

The Distribution of Co-Care Activities Scale was adapted into Chinese for the purposes of this study, and then the psychometric characteristics of the Chinese version of the DoCCA scale were confirmed in chronic conditions.

**Methods:**

A total of 434 patients with chronic diseases were recruited from three Chinese cities. A cross-cultural adaptation procedure was used to translate the Distribution of Co-Care Activities Scale into Chinese. Cronbach's alpha coefficient, split-half reliability, and test-retest reliability were used to verify the scale's reliability. Content validity indices, exploratory factor analysis, and confirmatory factor analysis were used to confirm the scale's validity.

**Results:**

The Chinese DoCCA scale includes five domains: demands, unnecessary tasks, role clarity, needs support, and goal orientation. The S-CVI was 0.964. Exploratory factor analysis yielded a five-factor structure that explained 74.952% of the total variance. According to the confirmatory factor analysis results, the fit indices were within the range of the reference values. Convergent and discriminant validity both met the criteria. Also, the scale's Cronbach's alpha coefficient is 0.936, and the five dimensions' values range from 0.818 to 0.909. The split-half reliability was 0.848, and the test-retest reliability was 0.832.

**Conclusions:**

The Chinese version of the Distribution of Co-Care Activities Scale had high levels of validity and reliability for chronic conditions. The scale can assess how patients with chronic diseases feel about their service of care and provide data to optimize their personalized chronic disease self-management strategies.

## 1. Introduction

Chronic disease is the leading cause of death and disability worldwide (1). In traditional care, professionals are the dominant figure in the interactive relationship; they are the experts who advise patients on what they should do (2). They may have unrealistic expectations about how much patients with chronic conditions can alter their behavior because they were largely trained in acute care. This is insufficient to meet the patients' requirements (3), resulting in failing to adequately safeguard patients' quality of life and bringing potential risks to their lives and health, even if the chronic disease ultimately has a regrettable outcome. Here, it is especially crucial for patient engagement in managing their chronic diseases. This requires a certain level of self-care on the part of patients during the process of chronic disease self-management (CDSM). Because of things like the disease characteristics and the upbringing, there are variable levels of self-care and support needs (4, 5). Therefore, it is urgently necessary for both medical and non-medical support to be individualized and precise in order to meet their different supportive needs, enhance their self-care abilities, and further promote their CDSM.

To facilitate patients in achieving their CDSM goals, it is necessary to develop a patient-centered model of healthcare service (6). Co-care is at the core of this type of strategy. In other words, the patients and the professionals work together to manage chronic diseases, with the patients as the experts in living and the professionals as the experts in the disease. Co-care is defined as an interaction system between patients and their resources, professionals and their resources, and information and communication technologies as tools (7). It aims to achieve a patient-valued and professional-endorsed health outcome. It emphasizes patient-centeredness, and the role of the professionals is to perfect the deficiencies for patients, combining both resources to reach the best achievable outcomes. Patients gain problem-solving skills to complete daily care activities in this system. There are significant advantages to co-care: first, it meets individualized needs while improving the quality of care; then, it creates good health outcomes; in addition, it leverages the human resources (including knowledge, skills, experience, etc.) of patients and their relatives; and furthermore, it saves medical and social resources, reduces the burden of care, and maximizes cost-effectiveness.

Patient-centered strategies have a positive effect on the CDSM. Patients may benefit from support and help from the strategies at the individual, organizational, and community levels (8). For instance, patients' physical, psychological, and social needs are met by empowering them (9–11). Group storytelling, online support groups, and other forms of group members assisting each other to improve patient care (12–14). Strong stakeholder support systems for patients through community engagement approaches to achieve sustainable health effects (15, 16). However, over 80% of strategies focus on developing the patient's knowledge and skills to achieve the goals set by the individual, including effective problem-solving strategies (17). Although patient-centered strategies are studied, they mostly ignore patients' experience. Poor experience undermines patients' confidence in their ability to improve their lives, i.e., self-efficacy, and hinders the production of better health outcomes.

Although instruments exist to assess experience, current instruments tend to focus on one part of the co-care system and have not been seen to assess the individual's experience of a system that incorporates all co-care activities as a whole. Examples include physician assessments of decision-making process perceptions and patient assessments of their interactions with health care professionals (18, 19). Therefore, von Thiele Schwarz et al. (20) drew on the theory of distributed cognition to develop the Distribution of Co-Care Activities (DoCCA) Scale. The theory of distributed cognition assumes that individuals interact with others and social technologies to achieve specific goals (21). This is similar to the concept of co-care. In chronic disease care management, patients and professionals use ICT to maximize the strengths of their respective resources and ultimately achieve health goals that satisfy them both.

The development of the DoCCA scale stems from how co-care activities are experienced as a whole. Co-care reflects the perspective of the system as defined by the individuals who were in it (22). That is, how care activities are optimally allocated should be defined by the patients in the system. Co-care, a distributed system of activities used in the management of chronic diseases, should be focused on the patients' cherished goals and support their needs to make significant progress toward those goals (7). Analyzing whether task requirements are available, task division is appropriate, and roles are specified is referred to as activity. Needs support refers to the degree to which patients feel that their needs are being met, no matter what the source. Goal orientation describes how well health care is adequately informed about and involved in helping patients achieve their desired goals. Patients with chronic diseases have decisions that affect their health all the time. Care activities can be distributed in different ways among patients, others, and related technologies, and only the patient can decide which distribution is most satisfying to him or her, which is what the DoCCA scale is designed to assess. It is not concerned with one part of the system, but rather assesses the patient's experience of the system as a whole.

Despite the patient-centered CDSM model's significant implications, it is still not widely used in China. In particular, there are no instruments available to assess how satisfied patients with chronic diseases are with the distribution of co-care activities. Therefore, the English DoCCA scale was adapted into Chinese via the process of cross-cultural adaptation in this research. Furthermore, the psychometric properties of the Chinese DoCCA scale were examined in the context of primary health care in China.

## 2. Methods

### 2.1. Study design and participants

From June 2022 to September 2022, three cities in Liaoning Province, China—Shenyang, Anshan, and Jinzhou—were the sites of the multicenter cross-sectional study. The factor analysis procedure's general rules, which call for a minimum of 10 respondents per item, were used to establish the sample size (23). However, a larger sample is preferred. To guarantee the validity of exploratory factor analysis and confirmatory factor analysis in this research, a minimum of twenty respondents per item was necessary. With the aid of community staff, 434 patients with chronic diseases were recruited using convenience sampling. Patients with chronic diseases who volunteered to participate in the study and were at least 18 years old were required to meet the inclusion criteria. Chronic conditions here were defined as hypertension, chronic heart failure or mental health problems (including reactions to severe stress and adjustment disorders, insomnia, anxiety disorders and depression). People who couldn't communicate, such as those with cognitive dysfunction and language impairment, were excluded.

### 2.2. Instruments

#### 2.2.1. General demographic characteristics questionnaire

By combining the pre-investigation information with the current literature, a general demographic information questionnaire was designed. This questionnaire had six aspects: age, sex, educational degree, income per month (RMB), number of diseases, and duration of disease (year). Participants who had chronic conditions were asked to self-report.

#### 2.2.2. Distribution of Co-Care Activities Scale

The Distribution of Co-Care Activities (DoCCA) Scale, created by von Thiele Schwarz et al. (20), was used to assess the experience of patients with chronic conditions in their care system. This scale's 20 items are all scored on a 5-point Likert response scale, with 1–5 representing very low, low, partial, high, and very high levels of accomplishment, respectively. Schwarz's team tested four alternative models, after which two acceptable models were obtained. One was a single-order five-factor model, where the model included five independent factors: demands, unnecessary tasks, role clarity, needs support, and goal orientation. The other was a second-order model, which included three factors: activities, needs support, and goal orientation; activities were divided into three subfactors: demands, unnecessary tasks, and role clarity. Schwarz's team chose the second-order model due to its better adaptation to the theory of distributed cognition. The scores are between 20 and 100. The patient has better experience with their chronic care system when the total score is higher. The Cronbach's alpha coefficients corresponding to the five factors of demands, unnecessary tasks, role clarity, needs support, and goal orientation were 0.92, 0.79, 0.90, 0.93, and 0.91, respectively.

### 2.3. Procedure

#### 2.3.1. Scale translation procedure

**Figure 1:**
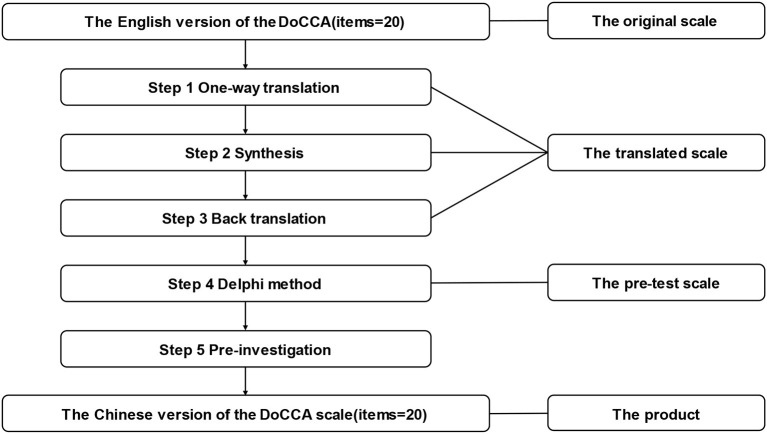
Scale translation procedure for Chinese DoCCA scale.

Schwarz gave authorization via email. Firstly, a Chinese professor with a specialty in English and a second Chinese professor with a specialty in chronic disease management translated the DoCCA Scale from English into Chinese. Secondly, the differences between the two Chinese translations were combined and processed by the group members. Thirdly, two native English speakers who were not familiar with the scale performed the back translation of it. Fourthly, the pre-test scale was created using two rounds of the Delphi method while comparing the equivalence of the translated scale and the original scale. There was a 2-week gap between the two rounds of the Delphi method. Psychology professors were also invited to adjust the translated scale. Fifthly, 15 patients with chronic diseases were recruited for the pre-investigation and asked if the layout of the scale was well designed and if the items were easily understood. The product is the Chinese version of the DoCCA scale (Figure 1).

#### 2.3.2. Data collection procedure

After making the necessary preparations, three groups were sent to three cities to recruit patients with chronic diseases with the help of community staff. Participants filled out the Chinese DoCCA scale independently and anonymously in a room provided by the community staff. If they had any questions, they could contact the group outside the house at any time. Of these, 40 patients with chronic diseases consented to retaking the questionnaire in 2 weeks and promised to continue using the same strategies for managing their diseases.

### 2.4. Data analysis

#### 2.4.1. Items analysis

The differences and significance of the two groups on each item were calculated using the top 27% of the overall score of the Chinese DoCCA scale as the high group and the bottom 27% as the low group. The correlation coefficients between each item and the scale were calculated, as well as the Cronbach's alpha coefficients after deleting each item. The three methods above were used to determine whether each item should be kept or not.

#### 2.4.2. Validity analysis

Seven experts from related fields were invited twice to assess the content validity of the translated scale through the Delphi method. In each round, all experts independently rated the content and structure of the scale and gave suggestions for modifications, which were used as strategies to adjust the scale. Either the content validity index of the item (I-CVI) or the content validity index of the scale (S-CVI) was used to assess whether the items on the translated scale were adequate and whether the proportion of content allocation was appropriate. The responses were scored on a 4-point Likert scale, with 1–4 representing no correlation, somewhat correlation, quite correlation, and high correlation. The I-CVI is the proportion of experts scoring 3 and 4 to all experts in each item. The I-CVIs for all items are averaged to create the S-CVI. In addition, face validity was assessed by study team members and participants during group discussions and the pre-investigation.

**Figure 2:**
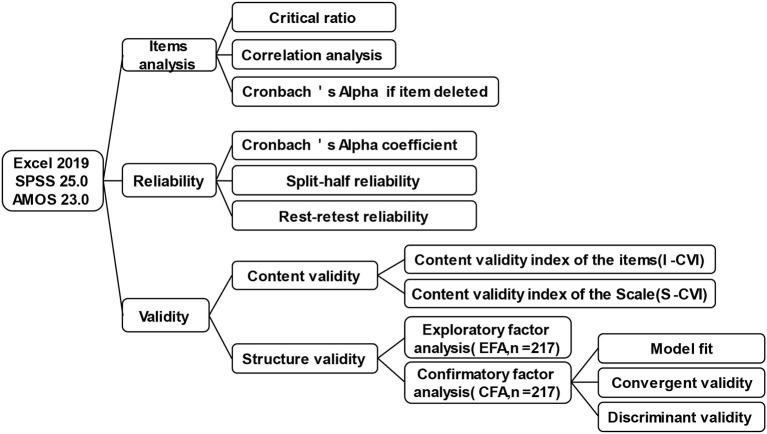
Data analysis procedure for Chinese DoCCA scale.

Either exploratory factor analysis (EFA) or confirmatory factor analysis (CFA) was employed to assess the factor structure of the Chinese DoCCA scale. The 434 patients with chronic diseases were split randomly into two groups: one group (*n* = 217) got EFA, and the other group (*n* = 217) got CFA. The basic characteristics of both groups were similar. It was decided whether the scale was reasonable for further factor analysis using the Kaiser–Meyer–Olkin (KMO) and Bartlett's test of sphericity. The best factor structure was obtained using principal component analysis (PCA) and variance maximization orthogonal rotation when Bartlett's test of sphericity was significant (*P* < 0.05) and the KMO was larger than 0.60. Based on the overall analysis of the visual inspection of the scree plot, eigenvalues, and total variance explanation, common factors were extracted. The Analysis of Moment Structure (AMOS) was applied to CFA to assess the appropriateness of the hypothesis model. Both convergent validity and discriminant validity were also analyzed (Figure 2).

#### 2.4.3. Reliability analysis

Two methods were used to gauge homogeneity and internal correlation among all the items. The Cronbach's alpha coefficients of the Chinese DoCCA scale were calculated in one and the split-half reliability of the scale using a before-and-after discount method in the other. Additionally, the Chinese DoCCA scale was employed in 40 patients with chronic conditions to calculate its test-retest reliability for the stability of the scale.

## 3. Results

### 3.1. Descriptive statistics

Four hundred thirty-four people with chronic diseases were included in the study. Over 65-year-old patients accounted for 36.2% of the total. 35.9% of the population was female, while 64.1% were male. 49.3 and 30.6%, respectively, of the participants had completed junior or senior secondary education. 41.0% of patients reported having a monthly income of RMB 3,000–4,000. Among the participants, only 12.7% had more than two chronic diseases. More than half of the patients (62.2%) had had chronic diseases for between 3 and 10 years. More detailed sociodemographic data is provided in Table 1.

**Table 1:** Frequency distribution of demographic characteristics (*n* = 434).

**Factors**	**Group**	***n***	**%**
Age	< 45	41	9.4
	< 55	97	22.4
	< 65	139	32.0
	≥65	157	36.2
Sex	Male	278	64.1
	Female	156	35.9
Educational degree	Primary schools or below	58	13.4
	Junior school	214	49.3
	Senior school	133	30.6
	College or above	29	6.7
Income per month (RMB)	< 2,000	83	19.1
	< 3,000	95	21.9
	< 4,000	178	41.0
	≥4,000	78	18.0
Number of illnesses	1	230	53.0
	2	149	34.3
	>2	55	12.7
Duration of illness (year)	< 3	86	19.8
	< 5	132	30.4
	< 10	138	31.8
	≥10	78	18.0

### 3.2. Adaptation and content validity analysis

For items 7, 8, 10, 11, 12, 17, 18, 19, and 20 of the original scale, a common word for healthcare was discovered during translation. This word has numerous meanings in Chinese, including healthcare, healthcare institution, healthcare professional, and healthcare system, among others. In order to address the mentioned problems, Prof. Schwarz sent an email to ask about the meaning of healthcare in several items. Healthcare refers to the healthcare system in items 7, 8, 10, 11, and 20 and to healthcare professionals in items 12, 17, 18, and 19. Some experts highlighted that the translation of items 4, 7, and 13 was inappropriate during the first round of the Delphi process. Following a group discussion, it was determined to modify items 4, 7, and 13 in accordance with professional advice and to make minor adjustments to items 1, 5, 15, and 16. The second round of the Delphi method produced satisfactory results. Furthermore, the pre-test scale received favorable feedback from 15 patients with chronic conditions.

Two rounds of the Delphi method were employed in this research. The I-CVI was 0.429–1.000 and the S-CVI was 0.886, according to the first Delphi method's findings. The I-CVI was 0.857–1.000 and the S-CVI was 0.964, according to the results of the second Delphi method (Table 2).

**Table 2:** Content validity analysis for Chinese DoCCA scale.

**Item**	**Experts (score) (first round/second round)**							**I-CVI (first round/second round)**
	**1**	**2**	**3**	**4**	**5**	**6**	**7**	
1	0/1	1/1	1/1	1/1	1/0	1/1	1/1	0.857/0.857
2	1/1	1/1	1/1	1/1	1/1	1/1	1/1	1.000/1.000
3	1/1	1/1	1/1	1/1	1/1	1/1	1/1	1.000/1.000
4	0/1	0/0	1/1	1/1	1/1	0/1	1/1	0.571/0.857
5	1/1	1/1	0/1	1/1	1/1	1/1	1/1	0.857/1.000
6	1/1	1/1	1/1	1/1	1/1	1/1	1/1	1.000/1.000
7	0/1	0/1	1/1	1/1	0/1	1/1	0/0	0.429/0.857
8	1/1	1/1	1/1	1/1	1/1	1/1	1/1	1.000/1.000
9	1/1	1/1	1/1	1/1	1/1	1/1	1/1	1.000/1.000
10	1/1	1/1	1/1	1/1	1/1	1/1	1/1	1.000/1.000
11	0/1	1/0	1/1	1/1	1/1	1/1	1/1	0.857.0.857
12	1/1	1/1	1/1	1/1	1/1	1/1	1/1	1.000/1.000
13	1/1	0/1	0/1	1/0	0/1	1/1	0/1	0.429/0.857
14	1/1	1/1	1/1	1/1	1/1	1/1	1/1	1.000/1.000
15	1/1	0/1	1/1	1/1	1/1	1/1	1/1	0.857/1.000
16	1/1	1/1	1/1	1/1	1/1	1/1	0/1	0.857/1.000
17	1/1	1/1	1/1	1/1	1/1	1/1	1/1	1.000/1.000
18	1/1	1/1	1/1	1/1	1/1	1/1	1/1	1.000/1.000
19	1/1	1/1	1/1	1/1	1/1	1/1	1/1	1.000/1.000
20	1/1	1/1	1/1	1/1	1/1	1/1	1/1	1.000/1.000

0, expert scores of 1 or 2; 1, expert scores of 3 or 4.

### 3.3. Items analysis

If the critical ratio (CR) of an item was more than 3.000, differences between high and low groups were regarded as adequate discrimination (24). The critical ratio (CR) in the study ranged from 12.657 to 21.492 for the 20 items. The scores for each item were correlated positively with the total score (*r* = 0.598–0.766, *P* < 0.001). After deleting each item, the Cronbach's alpha values for the Chinese DoCCA scale were 0.931–0.935, all of which were poorer than the total Cronbach's alpha value (0.936). Eventually, all 20 items of the Chinese DoCCA scale were finally retained (Table 3).

**Table 3:** Item analysis for Chinese DoCCA scale.

**Item**	**Item score (SD)**	**Critical ratio**	**Correlation coefficient**	**Cronbach's alpha if item deleted**
1	3.49 (1.20)	14.904	0.658	0.934
2	2.82 (1.16)	18.235	0.732	0.932
3	2.86 (1.15)	18.638	0.725	0.932
4	3.35 (1.15)	15.377	0.640	0.935
5	3.42 (0.93)	17.466	0.683	0.933
6	3.57 (0.99)	17.897	0.687	0.933
7	3.01 (0.94)	14.012	0.609	0.934
8	2.86 (0.85)	18.436	0.704	0.933
9	2.98 (0.85)	21.492	0.766	0.931
10	2.85 (0.86)	20.301	0.757	0.932
11	3.21 (0.86)	16.937	0.678	0.933
12	2.64 (0.81)	19.276	0.724	0.932
13	3.18 (0.76)	12.759	0.598	0.934
14	3.64 (0.66)	12.657	0.624	0.934
15	3.09 (0.82)	14.395	0.624	0.934
16	3.36 (0.77)	18.342	0.669	0.933
17	2.86 (0.84)	14.961	0.637	0.934
18	3.35 (0.86)	17.971	0.692	0.933
19	3.49 (0.84)	16.203	0.675	0.933
20	3.52 (0.82)	18.357	0.716	0.932

### 3.4. Structure validity analysis

#### 3.4.1. Exploratory factor analysis

**Figure 3:**
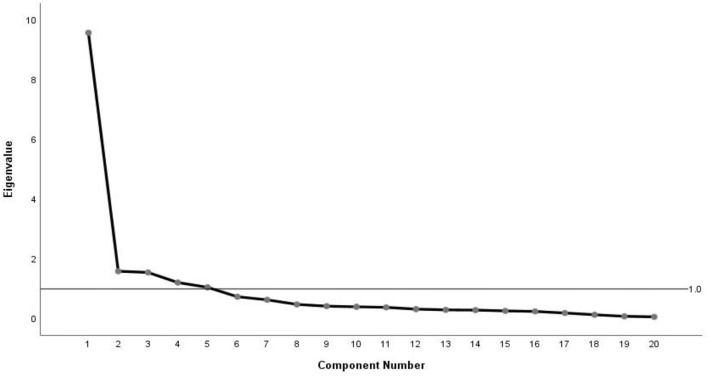
Screen plot of exploratory factor analysis for Chinese DoCCA scale.

The Bartlett's test of sphericity was significant (χ^2^ = 3,284.053; *P* < 0.001) and KMO > 0.6 (KMO = 0.908), indicating that the Chinese DoCCA scale was suitable for further extraction of the common factors and getting the best factor structure. It is commonly accepted that two distinct approaches can result in satisfactory structure validity: initially, the factors extracted by EFA must explain at least 40% of such total variance; and second, every item must have a high loading level for one factor (>0.400) and low loading levels for the others. In the research, the initial eigenvalues of all five factors were larger than 1 and explained 74.952% of the total variance; Meanwhile, every item's factor loading value exceeded 0.400. Furthermore, the five-factor structure was also verified by the scree plot (Figure 3). This was because the downward trend became weaker after the fifth point. The five factors respectively explained 18.182%, 15.957%, 14.538%, 13.992%, and 12.282% of the total variance after the variance maximization orthogonal rotation. The detailed factor loadings are shown in Table 4.

**Table 4:** Factor loadings of EFA for Chinese DoCCA scale.

**Item**	**Factor 1**	**Factor 2**	**Factor 3**	**Factor 4**	**Factor 5**
1	–	0.759	–	–	–
2	–	0.870	–	–	–
3	–	0.859	–	–	–
4	–	0.671	–	–	–
5	–	–	–	–	0.797
6	–	–	–	–	0.755
7	–	–	–	–	0.727
8	0.610	–	–	–	–
9	0.836	–	–	–	–
10	0.827	–	–	–	–
11	0.768	–	–	–	–
12	0.748	–	–	–	–
13	–	–	–	0.704	–
14	–	–	–	0.723	–
15	–	–	–	0.750	–
16	–	–	–	0.700	–
17	–	–	0.645	–	–
18	–	–	0.775	–	–
19	–	–	0.750	–	–
20	–	–	0.729	–	–

#### 3.4.2. Confirmatory factor analysis

**Figure 4:**
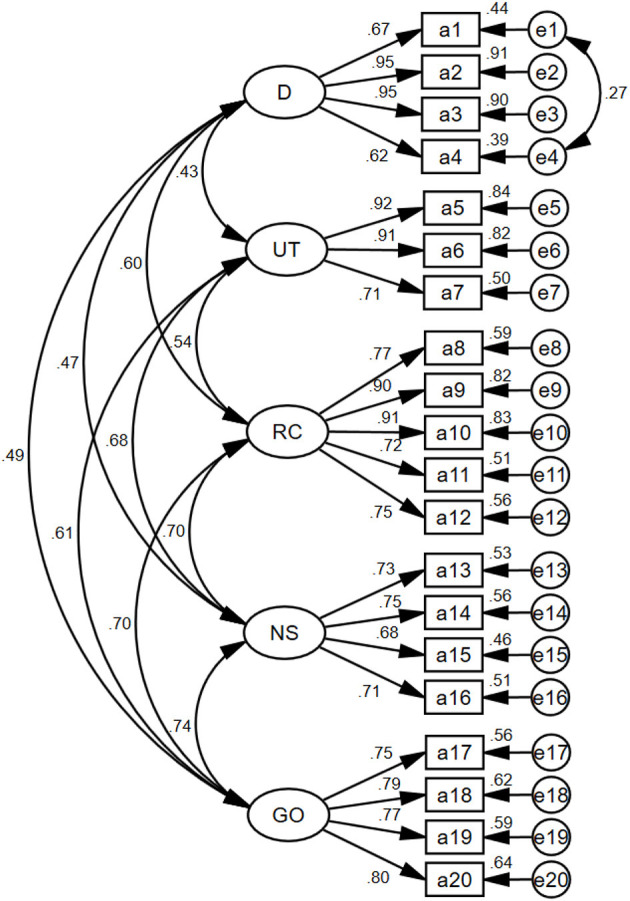
Standardized five-factor structural model of Chinese DoCCA scale (*n* = 217). D, demands, four items; UT, unnecessary tasks, three items; RC, role clarity, five items; NS, needs support, four items; GO, goal orientation, four items.

According to the Modification Index (MI), the initial model was revised once, i.e., e1 and e4. The fit indices both before and after adjustment are shown in Table 5. In the final model, the fit indices all met the criteria. The results of the model fit for the CFA are shown in Figure 4. The results for convergent validity and discriminant validity are shown in Table 6. The construct reliability (CR) values were 0.80–0.87 and the average variance extracted (AVE) values ranged from 0.52 to 0.63, demonstrating convergent validity (25). The correlation coefficients among the five factors were 0.482–0.626. And the square root of AVE for each factor was larger than the correlation coefficients between this factor and the other factors, demonstrating discriminant validity (25).

**Table 5:** Fit indices both before and after adjustment for Chinese DoCCA scale.

**Fit measures**	**Criteria**	**Fit index before modification**	**Fit index after modification**
GFI	≥0.900	0.917	0.925
AGFI	≥0.900	0.891	0.901
RMSEA	≤ 0.050	0.032	0.024
NFI	≥0.900	0.934	0.940
RFI	≥0.900	0.922	0.928
IFI	≥0.900	0.988	0.993
TLI	≥0.900	0.985	0.991
CFI	≥0.900	0.987	0.993
PGFI	≥0.500	0.699	0.700
PNFI	≥0.500	0.787	0.786
CMIN/DF	≤ 3.000	1.218	1.125

GFI, goodness-of-fit index; AGFI, adjusted goodness-of-fit index; RMSEA, root mean square error of approximation; NFI, normed fit index; RFI, relative fit index; IFI, incremental fit index; TLI, Tacker-Lewis index; CFI, comparative fit index; PGFI, parsimonious goodness-of-fit index; PNFI, parsimonious-adjusted normed-of-fit index; CMIN/DF, chi-square degree of freedom.

**Table 6:** Results of CFA for Chinese DoCCA scale.

**Factor**	**Correlation coefficient**					**AVE**	**CR**
	**D**	**UT**	**RC**	**NS**	**GO**		
D	0.794^*^					0.63	0.87
UT	0.482^**^	0.760^*^				0.58	0.80
RC	0.571^**^	0.597^**^	0.762^*^			0.58	0.87
NS	0.544^**^	0.493^**^	0.545^**^	0.720^*^		0.52	0.81
GO	0.525^**^	0.573^**^	0.609^**^	0.626^**^	0.726^*^	0.52	0.82

D, demands; UT, unnecessary tasks; RC, role clarity; NS, needs support; GO, goal orientation; CR, construct reliability.

^*^The square root of the AVE of this factor.

^**^P < 0.001.

### 3.5. Reliability analysis

The Cronbach's alpha value for the Chinese DoCCA scale was 0.936, and the five dimensions were 0.818–0.909. Additionally, the split-half reliability was 0.848. Two weeks later, 40 patients with chronic diseases were chosen for retesting, and the test-retest reliability was 0.832 (Table 7).

**Table 7:** Reliability analysis for Chinese DoCCA scale.

**The scale and its dimension**	**Score (SD)**	**Cronbach's alpha**	**Split-half reliability**	**Test-retest reliability**
The DoCCA	63.58 (12.33)	0.936	0.848	0.832
D	12.52 (4.04)	0.889		
UT	10.00 (2.52)	0.854		
RC	14.54 (3.62)	0.909		
NS	13.28 (2.43)	0.818		
GO	13.23 (2.83)	0.868		

D, demands; UT, unnecessary tasks; RC, role clarity; NS, needs support; GO, goal orientation.

## 4. Discussion

To the best of our knowledge, this is the first research in China to validate DoCCA in patients with chronic diseases. The instrument has proven to be reliable and valid for Chinese chronic conditions.

In this research, the convenience sampling method was used to select participants, which can cause some limitations. The sample was diverse in terms of socio-demographic background. Nonetheless, the recruitment process relied on staff working in community management who assisted in recruiting patients who were interested in this study and willing to talk about their own disease management. The participants were drawn from communities in one provincial capital city and two non-capital cities, but no indicators were used to ensure an appropriate balance between the various groups. Furthermore, the Chinese DoCCA scale includes only those who can have an effective conversation and does not include those who have language impairments for some reason. Future research should concentrate on these populations to ensure that all people with chronic conditions have the necessary tools to assess their experience with the distribution of co-care activities.

A measurement instrument's validity is defined by how closely it resembles the concept it is expected to study (26). Content validity is used to test the comprehensiveness, objectivity, and simplicity of the measurement instrument to determine whether the content of the scale meets the study objectives. The DoCCA scale was cross-culturally adapted using a stringent procedure—the AAOS recommended guidelines—resulting in the Chinese scale in the study (27). This approach ensured the content, semantic, technical, criterion, and conceptual equivalence between the original scale and the Chinese DoCCA scale (28). According to the Delphi method's discoveries, the I-CVI and the S-CVI were both above the reference levels (29). Additionally, the pre-investigation revealed that the Chinese DoCCA scale contained clear concepts, reasonable structure, and easily understandable content.

Structural validity is used to test whether the scale structure conforms to the target structure. In this research, the Chinese DoCCA scale was found to contain five factors through EFA, namely the demands, unnecessary tasks, role clarity, needs support, and goal orientation. This hypothetical structural model was again tested with the help of AMOS, and both results showed that this five-factor model was very good. Amusingly, however, this was both consistent and inconsistent with the findings of Schwarz's team. Compared to the original scale, the total number of items and the items under the same factor in the Chinese DoCCA scale remained unchanged, but the relative structure of the five factors was changed. Among the second-order model and the single-order five-factor model, the Schwartz team chose the former according to the theoretical framework. In light of this, further exploration of the Chinese DoCCA scale was considered, i.e., whether there was a higher-order latent variable (activities) influencing the three first-order factors (demands, unnecessary tasks, and role clarity). However, it was clear that this was not feasible, as the low two-by-two correlation of the three variables demands, unnecessary tasks, and role clarity showed that the three variables were directly independent. In addition, favorable convergent and discriminant validity demonstrated that the five factors of the Chinese DoCCA scale could be distinguished and that items that should fall under the same factor did fall under the same factor when measured.

The Chinese DoCCA scale is a single-order five-factor scale, indicating that the five factors are both relatively independent and work together to reflect the distribution of co-care activities. Compared with the original scale, the correlation of the three factors of demands, unnecessary tasks and role clarity in the Chinese DoCCA scale is not strong enough to jointly reflect the latent variable of activity for the following four possible reasons. First, the historical traditions and cultural differences between China and the West. In China, influenced by the Confucian “family sentiment” and the culture of sharing diseases in the family, family members will take over the task of chronic disease management for the patients, and the perceived demands of patients may not be sensitive. Second, the level of economic development is different. The chronic disease management model in China is far less mature than in Switzerland, and it may be that patient roles are generally not clear enough. Third, the participants were mostly over 55 years of age, who were socially experienced and had personal and unique perceptions and opinions about things but were more stubborn in their thinking and were not easily changed, even though bias may exist. Fourth, despite the sufficiently large sample size and the diversity of sociodemographic information in this research, a non-representative sample obtained through convenience sampling was used, which could potentially bias the results. In addition to this, in the context of chronic disease management in China, our aim was to assess the general factors of the distribution of co-care activities rather than to differentiate the effect of activities on the distribution of co-care activities or its relationship with other variables.

Reliability accurately reflects the true state of participants and is used to estimate the degree of stability and consistency of a measurement instrument (25). Cronbach's alpha and split-half reliability are used to assess scale homogeneity and internal correlation. The Cronbach's alpha coefficient of the Chinese DoCCA scale was >0.8, and each dimension was >0.6, which was also similar to the results of the original scale (18); meanwhile, the split-half reliability was high, both of which indicated that it had strong internal consistency. Additionally, test-retest reliability is used to measure scale stability across time. The retest reliability results of the Chinese DoCCA scale indicated that its temporal stability was in a very advantageous position.

In the process of co-care for chronic diseases, the optimal distribution of care activities is most appropriately determined by the patient as a client. A good patient experience enhances their wellbeing and intrinsic motivation and promotes positive self-management. However, there are relatively few studies in China that focus on patients' experience of co-care systems in chronic conditions, and one of the main reasons is the lack of a localized measurement instrument. In this research, the Chinese DoCCA scale containing 20 items was finally revised after one-way translation, back translation, the Delphi method, and pre-investigation, and its results had good reliability and validity. At the same time, the scale is simple and easy to understand for patients with chronic diseases, and it takes 3–5 min to fill in, which is highly operable. It reflects the patients' experience with co-care systems without adding to the burden of chronic disease patients.

Patient self-care is essential in CDSM. However, non-professional patients often need the assistance and support of healthcare professionals, a partnership that is often dominated by professionals and is highly susceptible to poor care at the expense of the patient experience. This is where having a tool to assess the experience of patients with chronic conditions is critical. Firstly, the Chinese DoCCA scale can be utilized to evaluate the comprehensive patient experience with the chronic management system rather than just a component of it. A satisfactory co-care system has low demands, few unnecessary tasks, clear roles, adequate needs support, and a patient-centered action orientation. This assists in improving the patients' quality of life. Further, the Chinese DoCCA scale can be used to compare interventions designed to improve CDSM. It reveals how changes in care delivery have improved patient experience. This helps optimize the chronic care system. Finally, the Chinese DoCCA scale can be employed to detect changes in patient preferences when used in conjunction with other interventions. It assists in developing care distribution strategies that most closely match patients' preferences. This contributes a significant amount to disease management and healthcare.

As summarized above, the Chinese DoCCA scale can be used in chronic conditions to assess patients' personal experience with the Chinese co-care system.

### 4.1. Limitations

This research has a few shortcomings that need to be noticed. These data were obtained from patients' self-reports; therefore, any biases that might exist should be taken into account. Furthermore, when using PCA to extract common components, the results might be overstated. Lastly, despite the fact that the sample size in this research was sufficient, the generalizability of these discoveries is limited by the convenience sampling.

## 5. Conclusions

With cross-cultural adaptation and psychological characteristic validation, this Chinese version of the DoCCA scale showed unexpected reliability and validity. It has now succeeded in being introduced to China. It will be used in community practice to provide information that will assist in developing the most suitable personalized CDSM strategies.

## Data availability statement

The raw data supporting the conclusions of this article will be made available by the authors, without undue reservation.

## Ethics statement

The studies involving human participants were reviewed and approved by the Medical Ethics Committee of the Jinzhou Medical University (JZMULL2022023). The patients/participants provided their written informed consent to participate in this study.

## Author contributions

MZ and HZ completed the study design. MZ, ML, QL, BH, YY, and SP performed material preparation and data collection. MZ and ML conducted the data analysis. MZ and QL interpreted the results. MZ drafted the manuscript. HZ edited and approved the manuscript. All authors commented on previous versions of the manuscript and approved the final manuscript.
